# Generation of a Yeast Cell Model Potentially Useful to Identify the Mammalian Mitochondrial *N*-Acetylglutamate Transporter

**DOI:** 10.3390/biom13050808

**Published:** 2023-05-10

**Authors:** Ruggiero Gorgoglione, Roberta Seccia, Amer Ahmed, Angelo Vozza, Loredana Capobianco, Alessia Lodi, Federica Marra, Eleonora Paradies, Luigi Palmieri, Vincenzo Coppola, Vincenza Dolce, Giuseppe Fiermonte

**Affiliations:** 1 Department of Bioscience, Biotechnology and Environment, University of Bari, 70125 Bari, Italy; 2 Department of Biological and Environmental Sciences and Technologies, University of Salento, 73100 Lecce, Italy; 3 Department of Nutritional Sciences, College of Natural Sciences, The University of Texas at Austin, Austin, TX 78712, USA; 4 Dell Pediatric Research Institute, Dell Medical School, The University of Texas at Austin, Austin, TX 78723, USA; 5 Department of Pharmacy, Health and Nutritional Sciences, University of Calabria, 87036 Arcavacata di Rende, Italy; 6 CNR Institute of Biomembranes, Bioenergetics and Molecular Biotechnologies (IBIOM), 70125 Bari, Italy; 7 Department of Cancer Biology and Genetics, College of Medicine, The Ohio State University and Arthur G. James Comprehensive Cancer Center, Columbus, OH 43210, USA

**Keywords:** *N*-acetylglutamate, urea cycle, mitochondrial carriers, yeast cell model

## Abstract

The human mitochondrial carrier family (MCF) consists of 53 members. Approximately one-fifth of them are still orphans of a function. Most mitochondrial transporters have been functionally characterized by reconstituting the bacterially expressed protein into liposomes and transport assays with radiolabeled compounds. The efficacy of this experimental approach is constrained to the commercial availability of the radiolabeled substrate to be used in the transport assays. A striking example is that of *N*-acetylglutamate (NAG), an essential regulator of the carbamoyl synthetase I activity and the entire urea cycle. Mammals cannot modulate mitochondrial NAG synthesis but can regulate the levels of NAG in the matrix by exporting it to the cytosol, where it is degraded. The mitochondrial NAG transporter is still unknown. Here, we report the generation of a yeast cell model suitable for identifying the putative mammalian mitochondrial NAG transporter. In yeast, the arginine biosynthesis starts in the mitochondria from NAG which is converted to ornithine that, once transported into cytosol, is metabolized to arginine. The deletion of ARG8 makes yeast cells unable to grow in the absence of arginine since they cannot synthetize ornithine but can still produce NAG. To make yeast cells dependent on a mitochondrial NAG exporter, we moved most of the yeast mitochondrial biosynthetic pathway to the cytosol by expressing four *E. coli* enzymes, *argB-E*, able to convert cytosolic NAG to ornithine. Although *argB-E* rescued the arginine auxotrophy of *arg*8∆ strain very poorly, the expression of the bacterial NAG synthase (*argA*), which would mimic the function of a putative NAG transporter increasing the cytosolic levels of NAG, fully rescued the growth defect of *arg*8∆ strain in the absence of arginine, demonstrating the potential suitability of the model generated.

## 1. Introduction

Mitochondrial carriers (MCs) are a family of eukaryotic intracellular transport proteins, primarily localized in the inner mitochondrial membrane, characterized by a tripartite structure consisting of three homologous domains, each composed of approximately 100 amino acids and containing a signature sequence motif [1,2,3]. By shuttling metabolites and co-factors across the inner mitochondrial membrane, MCs are master regulators of many metabolic pathways, especially those occurring partly in the matrix and partly in the cytoplasm. The mammalian urea cycle (UC) is a crucial example requiring at least three different MCs for correct functioning. Two of them, the ornithine carrier (ORC) [4,5] and the aspartate/glutamate carrier (AGC) [6,7,8,9], have already been functionally characterized in various organisms. The ORC exchanges cytosolic ornithine for mitochondrial citrulline; the former is used by the matrix carbamoyl synthetase I (CPSI) to synthesize citrulline, and the latter, once transported to the cytosol, is used with aspartate for the synthesis of argininosuccinate catalyzed by the argininosuccinate synthase. Humans have two ORC isoforms encoded by two different genes, SLC25A15 (ORC1) and SLC25A2 (ORC2). Mutations in the SLC25A15 gene lead to a rare recessive disorder named HHH (hyperornithinemia–hyperammonemia–homocitrullinuria) syndrome [4,10,11]. The AGC carrier exchanges cytosolic glutamate for mitochondrial aspartate. In humans, the two AGC isoforms SLC25A12 (AGC1) and SLC25A13 (AGC2) are encoded by two different genes and expressed in a tissue-specific manner [12,13]. Although mammals have a specific mitochondrial aspartate exporter, UCP2 [14,15,16,17], it seems that the cytosolic aspartate required for the synthesis of argininosuccinate is provided by the exchange reaction catalyzed by AGC2, the liver-specific isoform, since mutations in SLC25A13 cause an adult form of citrullinemia (CTLN2) in humans [7,18]. The third not yet identified mitochondrial transporter involved in the UC performs the mitochondrial efflux of NAG. The UC in mammals is regulated mainly by the amounts of UC enzymes and the concentrations of NAG and ornithine [19]. The synthesis of NAG from acetyl-CoA and glutamate is catalyzed by the liver mitochondrial NAG synthase (NAGS). NAG is an essential allosteric activator of mitochondrial CPSI, the key enzyme of the UC. The deficiency of NAGS is biochemically and clinically indistinguishable from that of CPSI [20]. NAG mitochondrial concentration varies in relationship to ammonium concentration in diet [21,22]. Its matrix concentration is regulated by its efflux from mitochondria and subsequent hydrolysis to glutamate and acetate in the cytosol [23]. Although a carrier-mediated efflux of NAG in energized mitochondria was initially discovered in 1982 [24], the mitochondrial transporter responsible for this biochemical function is still unknown.

**Figure 1:**
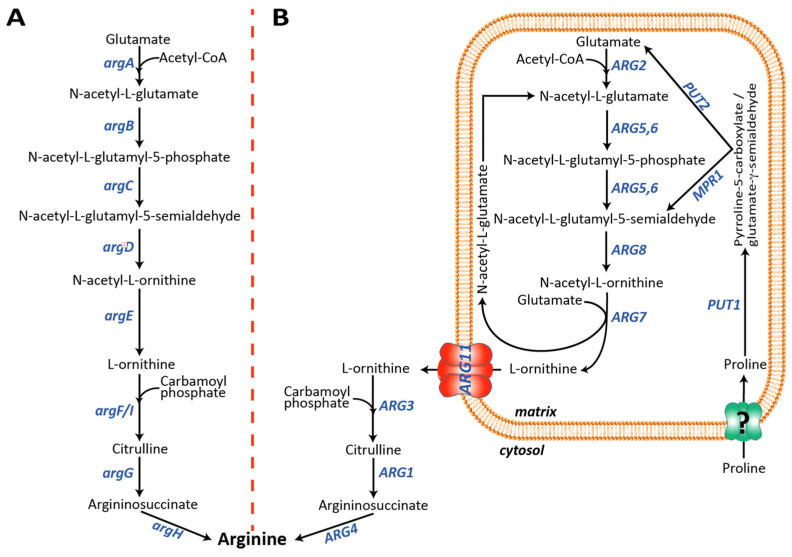
*Saccharomyces cerevisiae* and *Escherichia coli* arginine biosynthetic pathways. (**A**) In *E. coli*, arginine biosynthesis occurs in the cytosol. *argA*, NAG synthase; *argB*, NAG kinase; *argC*, *N*-acetyl-glutamyl-5-phospate reductase; *argD*, *N*-acetyl-ornithine amino transferase; *argE*, *N*-acetyl ornithine deacetylase; *argF/I*, ornithine carbamoyl transferase; *argG*, argininosuccinate synthase; *argH*, argininosuccinate lyase. (**B**) In *S. cerevisiae*, the arginine biosynthesis starts in the mitochondrial matrix from the condensation of glutamate with acetyl-CoA catalyzed by *ARG2* and ends with the synthesis of ornithine catalyzed by *ARG7*. Ornithine is then transported to the cytosol by *ARG11*, where it is finally converted to arginine. In *S. cerevisiae* the mitochondrial catabolism of proline may also produce *N*-acetyl-L-glutamyl-5-semialdehyde bypassing the first two steps of the main biosynthetic pathway. Although mechanistically the first three steps of the two pathways are not identical (see text for more details), the same metabolic intermediates are involved in both species. *ARG2*, NAG synthase; *ARG5,6*, *N*-acetylglutamate kinase and *N*-acetyl-5-glutamyl-phosphate reductase; *ARG8*, *N*-acetyl-ornithine aminotransferase; *ARG7*, *N*-acetyl-ornithine acetyltransferase; *ARG11*, mitochondrial ornithine transporter; *ARG3*, ornithine carbamoyl transferase; *ARG1*, argininosuccinate synthetase; *ARG4*, argininosuccinate lyase; *PUT1*, proline oxidase; *PUT2*, (S)-1-pyrroline-5-carboxylate dehydrogenase. *MPR1*, L-azetidine-2-carboxylic acid acetyltransferase.

Interestingly, NAG exerts distinct biological roles in lower compared to higher organisms. Differently from ureotelic vertebrates, in prokaryotes and lower eukaryotes, such as *Escherichia coli* and *Saccharomyces cerevisiae*, respectively, NAG is the first intermediate of the arginine biosynthesis (Figure 1) [25]. In both organisms, the arginine synthesis starts from the formation of NAG and has ornithine as an intermediate [26,27]. In *E. coli*, the arginine synthesis is a linear metabolic pathway catalyzed by eight genes, named *argA-H*, localized in the arginine operon (Figure 1A). *argA* synthesizes NAG, *argB-E* implement the generation of ornithine [26]. Although the UC does not occur in *S. cerevisiae*, the yeast arginine biosynthetic pathway is similar to the vertebrates’ UC since it occurs partly in the mitochondrial matrix and partly in the cytoplasm. The arginine biosynthetic pathway in *S. cerevisiae* requires nine different genes, *ARG1-8* and *ARG11* [27,28] (Figure 1B).

*N*-acetyl-glutamate synthase (Arg2p) and *N*-acetyl-ornithine acetyltransferase (*A*rg7p) synthesize NAG in the mitochondria. *N*-acetyl-ornithine acetyltransferase also catalyzes the formation of ornithine (Figure 1B), which, once transported to the cytoplasm by the mitochondrial ornithine carrier (Arg11p), is finally converted to arginine by Arg3p, Arg1p, and Arg4p. Although this is the main yeast arginine biosynthetic pathway, an alternative route has been proposed in which the (S)-1-pyrroline-5-carboxylate (P5C), or its spontaneous tautomer glutamate-γ-semialdehyde (GSA), derived from the mitochondrial proline catabolism, is converted to *N*-acetyl-glutamate-5-semialdehyde by *N*-acetyltransferase (Mpr1p) [29], bypassing the first three steps of the main pathway (Figure 1B). Most of the functionally characterized mitochondrial transporters have been identified by reconstitution of the bacterially expressed recombinant proteins into liposomes followed by transport assays carried out using radiolabeled substrates [9,30,31,32,33,34,35]. Two main issues can limit this experimental approach: (i) recombinant transporters, usually expressed as inclusion bodies, need to be refolded in an active form before their reconstitution into liposomes and protein folding may not occur properly; (ii) not all metabolites, known to cross the inner mitochondrial membrane, are available in a radiolabeled form. However, this latter limitation can sometimes be overcome by using chemically related molecules [36,37,38,39,40,41,42,43]. Indeed, the identification of the mitochondrial NAG transporter is negatively affected by the lack of commercially available radiolabeled NAG. In the present study, we set up a yeast cell model that can be used for identifying putative NAG carrier(s). The tool that we have engineered may be used to test the NAG transport activity of the remaining members of the mitochondrial carrier family or other mitochondrial transporter families that are still orphan of a function [44,45].

## 2. Materials and Methods

### 2.1. Yeast Strains and Growth Conditions

A BY4742 (wild-type, MATα his3Δ1 leu2Δ0 lys2Δ0 ura3Δ0) yeast strain was provided by the EUROFAN resource center EUROSCARF (Frankfurt, Germany). The yeast gene deletions (*arg2*∆/*arg7*∆ and *arg8*∆) were achieved as described before [9]. The *ARG2* and *ARG8* deletion cassettes, carrying the nourseothricin resistance gene, were amplified using the pAG25 plasmid as a template, whereas that of *ARG7*, carrying the kanamycin resistance gene, was amplified using the pUG6 plasmid as a template. Supplementary Table S1 lists all oligos used. Yeast cells were transformed using the lithium acetate method [46] and transformants were selected on synthetic complete medium (SC) plates lacking uracil (SC–URA) or uracil and histidine (SC–URA/HIS). All yeast strains were phenotyped on (SC–URA) or (SC–URA/HIS) plates in the presence or absence of arginine. Cultures were started from medium log precultures grown on SC selective media in the presence of arginine; then, cells were washed, diluted, and spotted on the selective SC media, with or without arginine, and incubated for 48 h at 30 °C. Ten-fold serial dilutions of wild-type, deleted, and transformed strains were analyzed.

### 2.2. Construction of the Multicopy Expression Vectors

To express up to five genes, we initially cloned various combinations of the genes of interest in the polycistronic yeast expression vector pSP-GM2 [47,48]. The five *E. coli* genes, *argA-E*, were amplified by PCR using the DNA of the TOP10 strain as a template [49] *argA*, *argC*, and *argD* were cloned by *Bam*HI/*Sal*I digestion under the control of the TEF1 promoter and carried a Myc-Tag at their C-*termini* (Supplementary Figure S1). *argB* and *argE*, carrying an HA-Tag at their C-*termini*, were cloned by *Spe*I/*Sac*I digestion under the control of the PGK1 promoter in the pSP-GM2_*argC* and pSP-GM2_*argD*, respectively (Supplementary Figure S1). All plasmids were sequence verified. Supplementary Table S1 lists all oligos and cloning sites used.

### 2.3. Construction of Centromeric Polycistronic Expression Vectors

**Figure 2:**
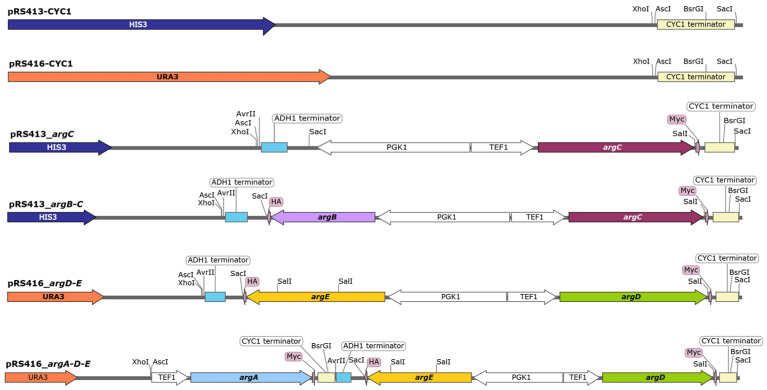
Schematic representation of the centromeric plasmid constructs. The pRS413-CYC1 and pRS416-CYC1 entry vectors were produced from the original centromeric vectors by cloning *Xho*I/*Sac*I the CYC1 terminator in the polylinker region. The presence of *Asc*I restriction site adjacent to *Xho*I allowed the further cloning of the whole expression cassettes already cloned in the pSP_GM2 plasmids. The pRS413_*argC*, pRS413_*argB-C*, and pRS416_*argD-E* constructs were generated by cloning *AscI*/*Bsr*GI the whole expression cassettes of the of the pSP-GM2 plasmids reported in Figure S1. The pRS416_*argA-D-E* plasmid was originated by cloning *Asc*I/*Avr*II the PCR-amplified fragment TEF1-*argA*-CYC1 (pSP-GM2_*argA*, Supplementary Figure S1) in the vector pRS416_*argD-E*. See details in the text.

To place the pSP-GM2 expression cassettes in the yeast centromeric vectors, the CYC1 terminator region of pSP-GM2 was amplified by PCR and cloned by *Xho*I/*Sac*I digestion into the polylinker of pRS413 or pRS416 vectors (Figure 2). Adjacent to the *Xho*I site, the forward primer carried an *Asc*I restriction site which permitted the further subcloning of the pSP-GM2 polycistronic expression cassettes in the two modified centromeric vectors. The whole polycistronic expression modules with part of the CYC1 terminator of pSP-GM2_*argC*, pSP-GM2_*argB-C*, and pSP-GM2_*argD-E* were digested by *Asc*I and *Bsr*GI and then subcloned in the modified pRS413 (*argC* and *argB-C*) or pRS416 (*argD-E*) vectors (Figure 2). The TEF1-ORF-CYC1 expression cassettes of pSP-GM2_*argA* was amplified by PCR and cloned *Asc*I and *Avr*II in the pRS416_*argD-E* (Figure 2).

The *ARG8*-pRS416, *ARG2*-pRS416 and *ARG7*-pRS413 plasmids were constructed by cloning DNA fragments of approximately 2200, 2700 and 2200 bp, containing in the centromeric plasmids the open reading frame and approximately 700 bp upstream and 250 bp downstream of the three genes, respectively. All plasmids were sequence verified. All oligos and cloning sites used are listed in Supplementary Table S1.

### 2.4. Western Blot Analysis

Yeast cell extracts were prepared as previously described [14,50]. Briefly, the various yeast strains were grown to 2–2.5 OD600 in liquid SC–URA/HIS and glucose as carbon source. The cell pellet was washed twice with distilled water and incubated in 0.1 M NaOH for 5 min at room temperature. The NaOH-treated cells were centrifuged at 12,000× *g* for 5 min, resuspended in a suitable volume of Laemmli sample buffer, and boiled for 3 min before loading. Yeast cell lysates (approximately 50 μg of proteins) were separated by SDS–polyacrylamide gel electrophoresis and transferred onto nitrocellulose membrane by a Trans-Blot Turbo Transfer System (Bio-Rad Laboratories, Rome, Italy). Membranes were blocked in Tris-buffered saline (TBS) containing 1% BSA and 0.1% Tween 20 before incubation with an anti-V5-Tag (1:5000 catalog no. 13,202; Cell Signaling Technology, Leiden, The Netherlands) or anti-HA.11 Epitope Tag Antibody clone 16B12 (1:5000 catalogue no. 901501; BioLegend, London, UK) primary antibody overnight at 4 °C. A rabbit antiserum against yeast Aac2p, a kind gift from L. Pelosi, was used for protein normalization [51,52]. Membranes were washed with TBS and Tween 20 and incubated with the appropriate dilution of the horseradish peroxidase (HRP)-conjugated secondary antibody for 1 h at room temperature and washed again [53,54,55]. The SuperSignal West Pico PLUS Chemiluminescent Substrate (no. 34,577; Thermo Fisher Scientific, Monza, Italy) was used for immunodecoration. Images were acquired by GelDoc (Bio-Rad Laboratories, Rome, Italy).

## 3. Results

### 3.1. ARG8 Deletion Induces an Arginine Auxotrophy in S. cerevisiae

**Figure 3:**
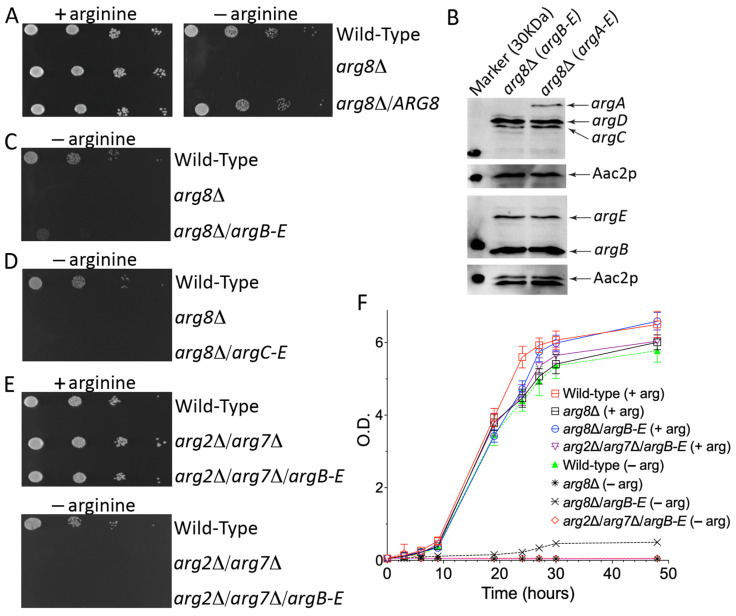
*arg8*∆ and *arg2*∆/*arg7*∆ mutants are auxotrophic for arginine. (**A**,**C**–**F**), Growth behavior of *arg8*∆ and *arg2*∆/*arg7*∆ mutants grown in SC medium in the presence or absence of arginine and glucose as sole carbon source. (**A**,**C**–**E**), Ten-fold serial dilutions of wild-type BY4742 cells and various yeast strains were plated. (**F**), The various yeast strains were inoculated in SC medium supplemented with 2% glucose in the presence or absence of arginine. The values of optical density at 600 nm refer to cell cultures after the indicated periods of growth. Data from three technical replicates are reported. Similar results were obtained in three independent experiments. (**B**), Expression levels of *argA-E* in the *arg8*∆ expressing *argB-E* or *argA-E* assayed with an anti-HA and anti-Myc tags specific antibodies (see text for more details). The loaded extracts were normalized by an anti-Aac2p antibody.

A suitable yeast cell model to identify the mammalian mitochondrial NAG transporter could be developed by blocking the arginine biosynthesis at the mitochondrial level and creating, at the same time, a novel NAG-dependent cytosolic pathway. The latter task could be easily achieved by expressing four *E. coli* enzymes (*argB-E)* which, starting from NAG, could synthesize ornithine in the cytosol. The former task needed to be investigated in more detail since we needed to block the arginine biosynthesis in the matrix without affecting the NAG synthesis, which, in the final model, had to be exported to the cytosol by the unknown transporter and used by *argB-E* for its conversion to ornithine. Among the mitochondrial enzymes involved in the arginine biosynthesis, the SGD database (https://www.yeastgenome.org/ (accessed on 21 April 2023)) reports that the single deletion of *ARG2* (YJL071w), *ARG5,6* (YER069w) and *ARG7* (YMR062c) genes leads to an arginine auxotrophy [56,57,58]. The deletion of *ARG2* or *ARG7* is not helpful for our project since both genes synthesize mitochondrial NAG (Figure 1). The deletion of *ARG5,6* is not functional as well because it is known that in yeast Arg5,6p forms a metabolon with Arg2p, and deletions of various length of the *ARG5,6* locus affects the enzymatic activity of Arg2p [59].We should also emphasize that performing a blast search of different databases, SGD included, using the human NAG synthase protein sequence, invariably produces the best score with the yeast Arg5,6p and not Arg2p (Supplementary Figure S2), suggesting that in the yeast metabolon, Arg5,6p may be directly involved with Arg2p in the NAG synthesis. For all these reasons, we focused our studies on the *ARG8* gene. On the one hand, deleting this gene would impair mitochondrial ornithine and arginine synthesis and increase the mitochondrial NAG levels. Furthermore, *ARG8* deletion would also cut the proline-derived *N*-acetyl-L-glutamyl-5-semialdehyde out. As shown in Figure 3A,F, the *arg8*∆ strain did not grow on the SC medium without arginine, whereas no growth defect was observed in the rescued strain with the heterologous expression of Arg8p or in the presence of arginine.

### 3.2. arg8∆ Strain Expressing argB-E Shows a Leaky Phenotype When Grown on SC Medium without Arginine

Although the results on the *arg8*∆ strain were encouraging, it was essential to verify that: (i) yeast does not express any endogenous NAG mitochondrial transporter, which would make our final model useless; (ii) the NAG synthesis in yeast occurs only in the matrix. To exclude both possibilities, we expressed four *E. coli* enzymes, *argB-E*, in the *arg8*∆ strain since, in the presence of cytosolic NAG, they could bypass the mitochondrial block generated by the lack of Arg8p.

The four genes were initially cloned in a multicopy plasmid (pSP-GM2) under the control of strong constitutive promoters (TEF1/PGK1) (Supplementary Figure S1). Yeast transformants expressing *argB-E* showed a different colony morphology (size and edge, not shown), suggesting that the expression of the four bacterial enzymes made the yeast strain unstable. A possible explanation could be that a high expression of the four *E. coli* proteins overworks the yeast protein synthesis apparatus. This issue was solved by moving all the expression cassettes from the pSP-GM2 to the centromeric pRS vectors (Figure 2). Although we observed poor but visible growth, the successful expression of the four bacterial enzymes (Figure 3B) did not fully rescue the growth defect of the *arg8*∆ strain when grown in the absence of arginine (Figure 3C,F). Since the four bacterial enzymes could partially restore the growth defect by using any intermediates from the NAG to NAG-γ-semialdehyde arginine biosynthetic pathway, we further verified the essential role of NAG in our model by expressing only *argC-E* in the *arg8*∆ strain. As shown in Figure 3D, the lack of bacterial *N*-acetylglutamate kinase (*argB)* made again the strain fully auxotrophic for arginine. This result clearly demonstrated that the cytosolic synthesis of ornithine in the *arg8*∆ strain expressing *argB-E* started from NAG and not from other downstream intermediates.

### 3.3. In Yeast the NAG Synthesis Takes Place Only in Mitochondria

Although the previous experiments suggested that NAG is present in the yeast cytoplasm, they did not test whether NAG originates in the cytosol or the mitochondrion. To answer this question, we constructed a double-deleted yeast strain lacking *ARG2* and *ARG7*, the only known genes able to synthesize NAG in the matrix (Figure 1B). As expected, the double-deleted strain did not grow on SC lacking arginine. No growth defect was observed in the presence of the amino acid (Figure 3E,F), suggesting that the lack of Arg2p and Arg7p leads to a shortage of ornithine required to finalize the arginine biosynthesis in the cytosol (Figure 1B). To test this hypothesis and exclude any cytosolic synthesis of NAG, we expressed the four *E. coli* genes, *argB-E*, in this strain because the synthesis of NAG in the mitochondrion would make their enzymatic activity useless. As shown in Figure 3E,F the expression of the four bacterial genes could not rescue the growth defect of the *arg2*∆/*arg7*∆ strain on the SC medium without arginine. These results demonstrate that yeast NAG synthesis occurred only in the mitochondrial matrix.

### 3.4. The Enzymatic Activity of argB-E Depends on the Cytosolic Levels of NAG

**Figure 4:**
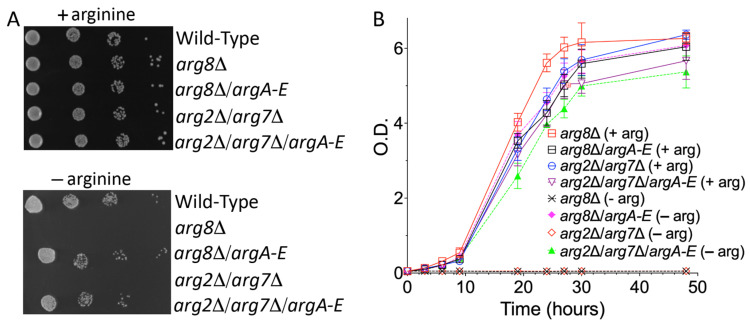
The expression of bacterial *argA-E* fully restored the arginine biosynthetic pathway in *arg8*∆ and *arg2*∆/*arg7*∆ mutants. Growth behavior of *arg8*∆ and *arg2*∆/*arg7*∆ mutants expressing the first five enzymes of the *E. coli* arginine biosynthetic pathway (*argA-E*) grown in SC medium in the presence or absence of arginine and glucose as sole carbon source. (**A**), Ten-fold serial dilutions of wild-type BY4742 cells and various yeast strains were plated. (**B**), The various yeast strains were inoculated in SC medium supplemented with 2% glucose in the presence or absence of arginine. The values of optical density at 600 nm refer to cell cultures after the indicated periods of growth. Data from three technical replicates are reported. Similar results were obtained in three independent experiments.

To prove that our model could be a valuable tool for identifying the putative mammalian mitochondrial NAG transporter, it was essential to understand the causes of the leaky growth showed by the *arg8*∆ strain expressing *argB-E* in the absence of arginine. There could have been at least two possible reasons. One is that yeast expressed an endogenous mitochondrial NAG transporter, and the four bacterial enzymes had poor enzymatic activity. The other is that the bacterial enzymes were fully functional and the leaking of NAG from mitochondria was due to its accumulation in the matrix which could induce a poor and non-specific transport by one or more than one mitochondrial transporter. In both cases, our model would be useless. To shed light on this point, we expressed in the *arg8*∆ and *arg2*∆/*arg7*∆ yeast strains the first five enzymes of the bacterial arginine biosynthetic pathway, *argA-E* (Figure 1), i.e., we also expressed the bacterial NAG synthase. As shown in Figure 4, the expression of the five bacterial enzymes fully rescued to proliferation defect in the absence of arginine in both yeast strains. These results confirmed that: (i) the NAG synthesis in yeast occurs only in the matrix; (ii) the cytosolic bacterial biosynthetic branch was fully functional; (iii) the model we generated was suitable to identify the putative mammalian NAG transporter.

## 4. Discussion

**Figure 5:**
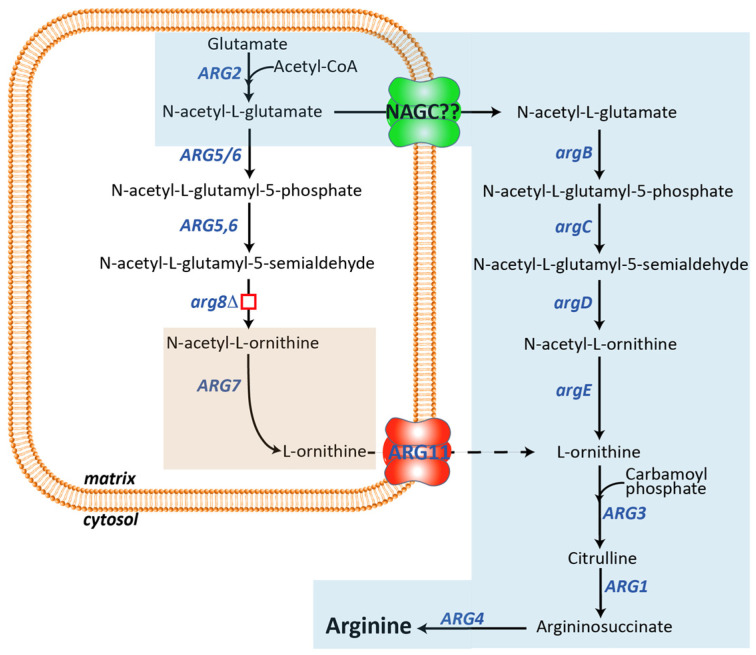
The yeast cell model generated to identify the putative mitochondrial NAG transporter/s. Yeast cells lacking the *ARG8* gene are auxotrophic for arginine. The expression of the *E. coli argB-E* in the cytosol makes cells dependent on the exit of NAG from the matrix. In this model, the expression of a functional unknown mitochondrial transporter able to catalyze the efflux of NAG from the matrix will make these cells able to grow in the absence of arginine. The part of the yeast mitochondrial arginine biosynthetic pathway not functional in the model is shaded brown whereas the newly created arginine biosynthetic pathway is shaded blue. *argB*, *argC*, *argD* and *argE*, *E. coli* NAG kinase, *N*-acetyl-glutamyl-5-phospate reductase, *N*-acetyl-ornithine aminotransferase and *N*-acetyl ornithine deacetylase, respectively; *ARG2*, NAG synthase; *ARG5,6*, *N*-acetylglutamate kinase and *N*-acetyl-5-glutamyl-phosphate reductase; *ARG8*, *N*-acetyl-ornithine aminotransferase; *ARG7*, *N*-acetyl-ornithine acetyltransferase; *ARG11*, mitochondrial ornithine transporter; *ARG3*, ornithine carbamoyl transferase; *ARG1*, argininosuccinate synthetase; *ARG4*, argininosuccinate lyase; NAGC??, putative mitochondrial NAG transporter.

The MCF belongs to the sizeable solute carrier protein (SLC) family, which consists of approximately 450 members [60]. The biochemical function of many members of the SLC family still needs to be discovered up to date. The leading cause of this need for more information is the difficulty of obtaining these proteins in a highly purified and active form suitable to be studied in artificial lipid vesicles (liposomes). A significant step forward in the field of MCF was made in 1993 when the first bacterial recombinant mitochondrial transporter was reconstituted in an active state into liposomes [61,62]. Since then, many other mitochondrial carriers have been functionally characterized by the same experimental approach. However, a significant number of them still need to be discovered. The functional identification of this group of proteins is of the utmost importance because their mutation can cause human diseases [32,63]. The biochemical characterization of these transporters facilitates their association with clinical alterations found in patients. To date, mutations in all enzymes and two mitochondrial transporters involved in the UC have been found (https://www.orpha.net/consor/cgi-bin/OC_Exp.php?lng=en&Expert=79167 (accessed on 21 April 2023)). The only exception is the mitochondrial NAG transporter, whose encoding gene is unknown. Although the NAG transporter is not directly involved in the UC, it has a key role in its regulation. Mammals cannot tightly control the mitochondrial synthesis of NAG and cannot degrade this metabolite in the matrix. When the UC must be downregulated, NAG is exported to the cytosol, where it is de-acetylated [23,24]. Currently, there is no suggested good candidate as mitochondrial NAG transporter among the remaining functionally uncharacterized members of the SLC families. In addition, the radiolabeled NAG, which is needed in transport assays with the recombinant reconstituted putative transporters, is not commercially available. Therefore, we aimed to set up an alternative yeast tool suitable for identifying the mitochondrial NAG carrier (Figure 5).

Taking advantage of the yeast and *E. coli* twin identical arginine biosynthetic pathways, we placed almost the entire yeast mitochondrial arginine biosynthetic pathway in the cytosol by expressing the bacterial *argB-E* enzymes. In this new yeast model, the biosynthesis of arginine depends on the efflux of NAG out of the mitochondrial matrix into the cytosol, where NAG is converted by the four bacterial enzymes to ornithine. This arrangement allows the bypassing of the block of mitochondrial synthesis of ornithine caused by the deletion of Arg8p. The expression of a carrier able to export NAG from mitochondria would make this yeast strain able to grow in the absence of arginine, as demonstrated in the yeast model expressing the bacterial enzymes *argA-E.* Furthermore, the data reported in Figure 3C,F and Figure 4 suggest that the yeast’s inner mitochondrial membrane contains endogenous transporters able to transport NAG, although not efficiently, probably when its concentration in the matrix increases due to the downstream block. To find the carrier(s) responsible for the NAG leak, we plan to delete several of yeast mitochondrial transporters, starting from those involved in the dicarboxylates transport [64,65,66]. Knowing the yeast NAG transporter(s) will be instrumental for identifying the putative orthologs in the higher eukaryotic up to humans. Furthermore, similar models could be generated to functionally characterize other mitochondrial carriers involved in the transport of extremely unstable substrates, which may produce artifacts in the transport assays.

## Data Availability

No new data were produced or analyzed in this study. Data sharing is not applicable to this article.

## Supplementary Materials

The following supporting information can be downloaded at: https://www.mdpi.com/article/10.3390/biom13050808/s1, Figure S1: Schematic representation of the multicopy plasmid constructs; Figure S2: Alignment of human NAGS with yeast ARG2 and ARG5/6; Table S1: Oligo sequences used.
