# Supplementary material for: Generation of a Yeast Cell Model Potentially Useful to Identify the Mammalian Mitochondrial N-Acetylglutamate Transporter
Source: Biomolecules. 2023 May 10;13(5):808. doi: 10.3390/biom13050808 (PMC10216676; doi:10.3390/biom13050808)
Supplement: Supplementary file 1 [file biomolecules-13-00808-s001.zip › Figures supplementary.zip!/Figures supplementary/Supplementary Table S1.pdf]

Supplementary Table S1	Oligo sequences used.	
Arg2_ΔF	TAGGACCGTCAATCTAGGACACTAGTCGGTTACTTTTATCAGCTGAAGCTTCGTACGCTGCAG	
Arg2_ΔR	AAAACTTGAAAGTTAAGGAGGAGGAGAATGCGTCTTTGTTGCATAGGCCACTAGTGGATCTGATATCAT	
Arg7_ΔF	TTCTATTCCCTTTATTTAAAGAAGTCCAAAATTCAACATAGCAGCTGAAGCTTCGTACGCTGCAG	
Arg7_ΔR	ACCATGCTTATAAAAGAAAGATTGAACAAAATATACATGAATGCATAGGCCACTAGTGGATCTGATATCAT	
Arg8_ΔF	GTTAGAATAATTCAAGAATCGCTACCAATCATGTTTAAAAGATAGCTGAAGCTTCGTACGCTGCAG	
Arg8_ΔR	CAATCTATACATGACAATTTACAAAGTATATTTAAGCGTAAACGCATAGGCCACTAGTGGATCTGATATCAT	
Arg2_pRS416_For	CGACGA**GGTACC**TTACCACGTAATTCGTGAATGGGGT	5' KpnI cloning site
Arg2_pRS416_Rev	CGACGA**GGATCC**GGCGCCTTACTTTGAGGAGAACG	3' BamHI cloning site
Arg7_pRS413_For	CGACGA**CTCGAG**CCACAATATGGATCATGCTAACCATTCTC	5' XhoI cloning site
Arg7_pRS413_Rev	CGACGA**GGATCC**GAGCACCAAGTCAGGTTAACATTACGTT	3' BamHiI cloning site
Arg8_pRS416_For	CGACGA**GGATCC**TTCGCTAGTATCAGCGGGTCTAGA	5' KpnI cloning site
Arg8_pRS416_Rev	CGACGA**CTCGAG**CTCATCATGTTAACACAGAAGGAAAAGTCA	3' XhoI cloning site
argA _ExFor	CGACGA**GGATCC**AACACAATGGTAAAGGAACGTAAAACCGAGTT	5' BamHI cloning site
argA _ExRev	CGACGA**GTCGAC**CCCTAAATCCGCCATCAACACT	3' SalI cloning site
argB _ExFor	CGACGA**ACTAGT**AACACAATGATGAATCCATTAATTATCAAACTGG	5' SpeI cloning site
argB _HA_ExRev	CGACGA**GAGCTC**TTAAGCATAATCTGGAACATCATATGGATAAGCTAAAATCCGCGTACCCAT	3' SacI cloning site
argC _ExFor	CGACGA**GGATCC**AACACAATGTTGAATACGCTGATTGTGGG	5' BamHI cloning site
argC _ExRev	CAGCGA**GTCGAC**AATAAGAGACTGCGTTTCCGCA	3' SalI cloning site
argD _ExFor	CGACGA**GGATCC**AACACAATGGCAATTGAACAAACAGCAAT	5' BamHI cloning site
argD _ExRev	CGACGA**GTCGAC**CGCCCCAACCACCTTCGCCAC	3' SalI cloning site
argE _ExFor	CGACGA**ACTAGT**AACACAAATGAAAAACAAATTACCGCCATTTAT	5' SpeI cloning site
argE _HA_ExRev	CGACGA**GAGCTC**TTAAGCATAATCTGGAACATCATATGGATAATGCCAGCAAAAATGGTGAATTA	3' SacI cloning site
argA _TEF1_ExFor	CGACGA**GGCGCGCC**CACACACCATAGCTTCAAAATGTTTC	5' AscI cloning site
argA_CYC1_ExRev	CGACGA**CCTAGG**CTTCGAGCGTCCCAAAACC	3' AvrII cloning site
CYC1_pRS_For	CGACGA**CTCGAG**GGCGCGCCATCCGCTCTAACCGAAAAGGAAG	5' XhoI/AscI cloning site
CYC1_pRS_Rev	CGACGA**GAGCTC**CTTCGAGCGTCCCAAAACCT	3' SacI cloning site
