# Supplementary material for: Generation of a Yeast Cell Model Potentially Useful to Identify the Mammalian Mitochondrial N-Acetylglutamate Transporter
Source: Biomolecules. 2023 May 10;13(5):808. doi: 10.3390/biom13050808 (PMC10216676; doi:10.3390/biom13050808)
Supplement: Supplementary file 1 [file biomolecules-13-00808-s001.zip › Figures supplementary.zip!/Figures supplementary/Supplementary Figure S2.pdf]

A

```
Query Length=534
                                                                          Score      E
Sequences producing significant alignments:                              (Bits)   Value

YER069W ARG5,6 SGDID:S000000871, Chr V from 295410-298001, Genome...    132     4e-33
YJL071W ARG2 SGDID:S000003607, Chr X from 306132-307856, Genome R...    47.8    3e-06
```

B

```
NAGS vs. ARG5/6

   Aligned Length = 540   Gapped Segments = 7   Total Mismatches (including gaps) = 430
   Identities = 110 (20.6%)   Similarities = 94 (17.6%)

NAGS      1 MATALMAVVLRAAAVAPRLRGRGGTGGARRLSCGARRRAARGTSPGRRLSTAWSQPQPPPEEYAGADDVSQSPVAEEPSWVPSPRPPVPHESPEPPSGRS 100
ARG5/6    1 MPSASLLVSTKRLNASKFQKFVSSLNKSTIAGFASVPLRAPPSVAFTRKKVGYSKRYVSS---TNGFSATRSTVIQLLNNISTKR-----EVEQYLKYFT  92
            * .* . *  .    .   .     . .      .    *  .    *     *.        ..    ..* * .    . . *     *  .     .

NAGS    101 LVQRDIQAFLNQCGASPGEARHWLTQFQTCHHSADKPFAVIEVDEEVLKCQQGVSSLAFALAFLQRMDMKPLVVLGLPAPTAPSGCLSFWEAKAQLAKSC 200
ARG5/6   93 SVSQQQFAVIKVGGAIISDNLHELASCLAFLYHVGLYPIVLHGTGPQVNGR------------LEAQGIEPDYIDGIRITDEHT----MAVVRKCFLEQN 176
             * .   * .   **   .  * *.   .          *.      .  .            *.   . *  . *.      .        .

NAGS    201 KVLVDALRHNAAAAVPFFGGGSVLRAAEPAPHASYGGIVSVETDLLQWCLESGSIPILCPIGETAARRSVLLDSLEVTASLAKALRPTKIIFLNNTGGLR 300
ARG5/6  177 LKLVTALEQLGVRARPITSGVFTADYLDKDKYKLVGNIKSVTKEPIEASIKAGALPILTSLAETASGQMLNVNADVAAGELARVFEPLKIVYLNEKGGII 276
              ** ** .    * *   *       .       *.* **  . .. .. .*..***. . ***. . . . .   **.   * **..**  **.

NAGS    301 D-SSHKVLSNVNLPADLDLVCNAEWVSTKERQQMRLIVDVLSRLPHHSSAVITAASTLLTELFSNKGSGTLFKNAERMLRVRSLDKLD-----QGRLVDL 394
ARG5/6  277 NGSTGEKISMINLDEEYDDLMKQSWVKYGTKLKIREIKELLDYLPRSSSVAIINVQDLQKELFTDSGAGTMIRRGYKLVKRSSIGEFPSADALRKALQRD 376
              *.   .* .**  . * .    **    . ..* * ..*  **. **  *     *  ***.  *.**. .   ....  *.         .  *

NAGS    395 VNASFGKKLRDDYLASLRPRLHSIYVSEGYNAAAILTMEPVLGGTPYLDKFVVSSSRQGQGSGQMLWECLRRDLQTLFWRSRVTNPINPWYFKHSDGSFS 494
ARG5/6  377 AGISSGKESVASYLRYLENSDFVSYADEPLEAVAIVKKD---TNVPTLDKFVCSDAAWLNNVTDNVFNVLRRDFPALQWVVSENDANIAWHFDKSQGSYL 473
             . * **     **  *       *  *   * **.  .    . * ***** * .   ..    .   ****  .* *    .     * * .* **.

NAGS    495 NKQWIFFWFGLADIRDSYELVNHAKGLPDSFHKPASDPGS 534
ARG5/6  474 KGGKVLFWYGIDDINTISELVENFVKSCDTASTLNSSAS  512
                . **.*. **    *** .     *.     *
```

C

```
NAGS vs. ARG2

   Aligned Length = 579   Gapped Segments = 13   Total Mismatches (including gaps) = 493
   Identities = 86 (15.0%)  Similarities = 88 (15.3%)

NAGS      1 MATALMAVVLRAAAVAPRLRGRGGTGGARRLSCGARRRAARGTSPGRRLSTAWSQPQPPPEEYAGADDVSQSPVAEEPSWVPSPRPPVPHESPEPPSGRS 100
ARG2      1 MWRRIFAHELKYDQPNASSK---NLILSVLNTTATKREAKDYLSKYTNDSGQHNHCLFFIRDLHKVAPAILSQFSSVIKRLGMLGLRPMFVIPPSPTHVN  97
            *   . *  *.        .   .   .   .. ..* *    *     *    .      .         *  .     .           *  *.

NAGS    101 LVQRDIQAFLNQCGASPGEARHWLTQFQTCHHS----ADKPFAVIEVDEEVLKCQQGVSSLAFALAFLQRMDMKPLVVLGLPAPTAPSGCLSFWEAK--- 193
ARG2     98 IQAELLDSIVTEADLKPLHLKEGLTKSRTGLYHSVFSQESRFFDIGNSNFIPIVKPYVYNEETASEFMTKDVVKFMDCLCQGNIPHIDKFFILNNAGGIP 197
            .    . . ....   *   .  **. .*         .  *  *     .   .  *     *  *. .  .* .  *                *

NAGS    194 -AQLAKSCKVLVDALRHNAAAAVPFFGGGSVLRAAEPAPHASYGGIVSVETDLLQWCLESG-------SIPILCPIGETAARRSVLLDSLEVTASLAKAL 285
ARG2    198 SGERNDNAHVFINLSQELEHLSSSLSHNISTLTKREPRSQNLLHRMEVYVKKDEISSLECEYHDHLENLLLMDKVLSNLAATATGLITTVKAAALSSDRK 297
              .    ..* .   .     .     . * *   **  .     .          .**.         . .   .   **  . *. ..  .*  .

NAGS    286 RPTKIIFLNNTGGLRD-SSHKVLSNVNLPADLDLVCNAEWVSTKERQQ---MRLIVDVLSRLPHHSSAVITAASTLLTELFSNKGSGTLFKNAERMLRVR 381
ARG2    298 NPLVYNLLTDRSLISSSLPRFKKKDGEIDSPANMFDDHAWYELPSQQVNAAPSNSDAVLVTTVLKKGVHIKTYDYKTLTQFNSIGLPKKFHVPEKGAKPS 397
             *     *.    .     .       . .   .     *     .*          **     .    * .        *   *    *.  *.  .

NAGS    382 S-LDKLDQGRLVDLVNASFGKKL-RDDYLASLRPRLHSIYVSEGYNAAAILTMEPVL-GGTPYLDKFVVSSSRQG-QGSGQMLWECLRRDLQ-TLFWRSR 476
ARG2    398 SNSPKLDINKFKSIIDQSFKRSLDLHDYIKRINGKIATIIVIGDYEGIAILTYEGSEENSFVYLDKFAVLPHLKGSLGISDIIFNLMFKKFPNEILWRSR 497
            *   *** ..   ..  ** . *   **.  . .. .* *   *   **** *    .   ***** *    .*  *   ..   . .     . ****

NAGS    477 VTNPINPWYFKHSDGSFSN-------------KQWIFFWFG--------LADIRDSYELVNHAKGLPDSFHKPASDPGS 534
ARG2    498 KDNVVNKWYFQRSVAVLDLSIDLDPEHCDEKQSQFKLFYYGNPQYAKRALRDKKRLREFMRSVRDIKPSWENEKNIS*  575
              * .* ***..*                    *   * .*        * * .   * .   . .  *
```

Supplementary Figure S2. Alignment of human NAGS with yeast *ARG2* and *ARG5/6*. A, The results of a BLASTP search of SGD database using the human NAGS as query sequence. B, C, ClustalW alignments of human NAGS with yeast *ARG5/6* and *ARG2*, respectively. Similarities and conservative substitutions are shown by asterisks and dots, respectively.
