# Supplementary material for: Generation of a Yeast Cell Model Potentially Useful to Identify the Mammalian Mitochondrial N-Acetylglutamate Transporter
Source: Biomolecules. 2023 May 10;13(5):808. doi: 10.3390/biom13050808 (PMC10216676; doi:10.3390/biom13050808)
Supplement: Supplementary file 1 [file biomolecules-13-00808-s001.zip › Figures supplementary.zip!/Figures supplementary/Supplementary Figure S1.pdf]

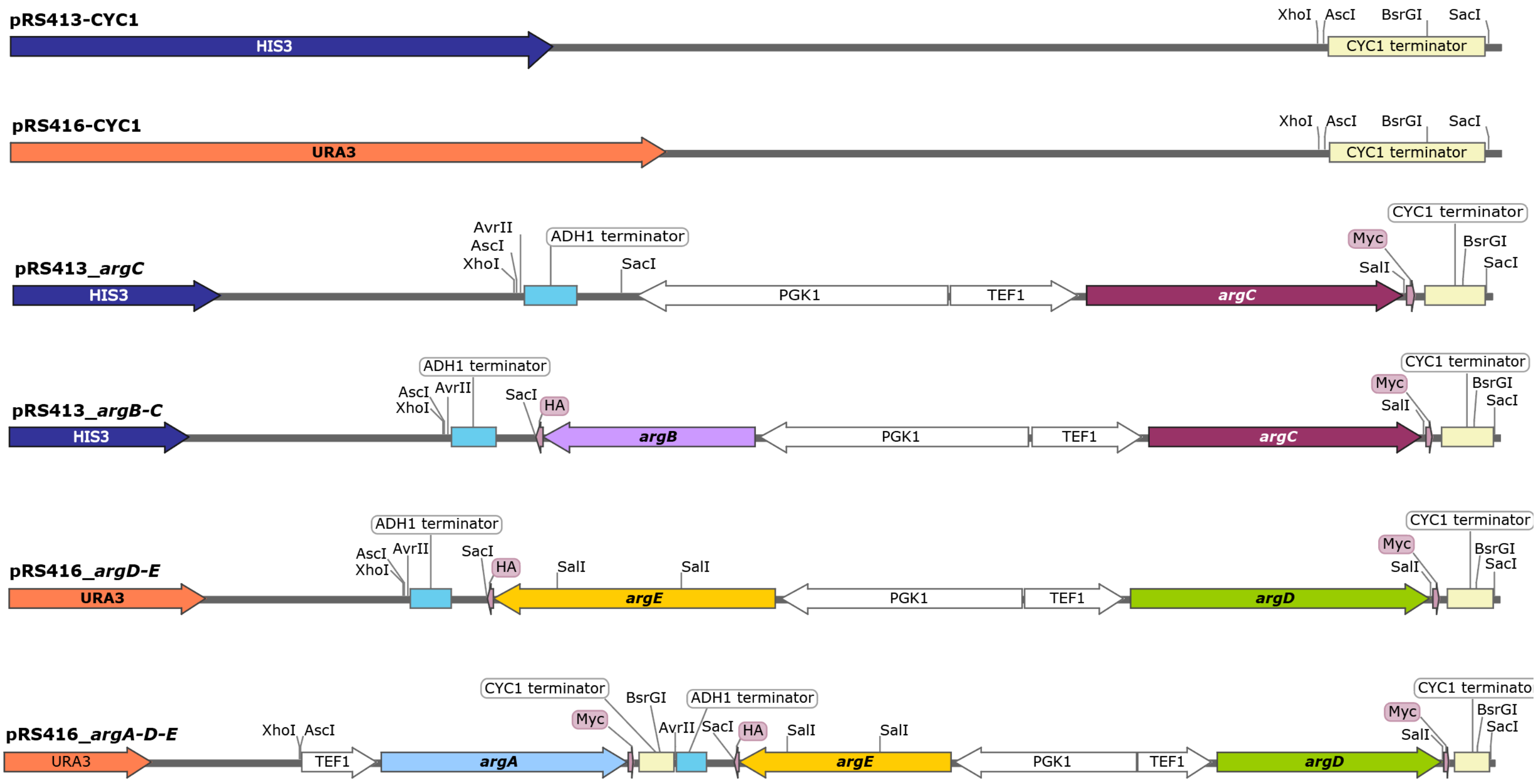

Supplementary Figure S1. Schematic representation of the multicopy plasmid constructs. The various pSP-GM2 plasmids containing the strong constitutive TEF1–PGK1 bidirectional promoter and expressing the *E. coli* enzymes (*argA-E*) are shown. All plasmids express the URA3 marker protein except the pSP-GM2_*argB-C* which expresses the HIS3 marker protein. The pSP-GM2 constructs carry two unique restriction sites, *Asc*I and *Avr*II, downstream the ADH1 terminator which allow the cloning of a third expression cassette. See details in the text.
